# Design, Fabrication, and Characterization of Capacitive Micromachined Ultrasonic Transducers for Transcranial, Multifocus Neurostimulation

**DOI:** 10.3390/mi15091106

**Published:** 2024-08-30

**Authors:** Tamzid Ibn Minhaj, Muhammetgeldi Annayev, Oluwafemi J. Adelegan, Ali Önder Biliroğlu, Feysel Yalçın Yamaner, Ömer Oralkan

**Affiliations:** 1 Department of Materials Science and Engineering, NC State University, Raleigh, NC 27695, USA; 2 Department of Electrical and Computer Engineering, NC State University, Raleigh, NC 27695, USA; mannaye@ncsu.edu (M.A.); ojadeleg@alumni.ncsu.edu (O.J.A.); abiliro@ncsu.edu (A.Ö.B.); fyyamane@ncsu.edu (F.Y.Y.); ooralka@ncsu.edu (Ö.O.)

**Keywords:** neurostimulation, capacitive micromachined ultrasonic transducer (CMUT), single-element, large area transducer, fabrication, design, FEM, non-invasive, ultrasound, nonhuman primate model

## Abstract

In a recent study using 3-D fullwave simulations, it was shown for a nonhuman primate model that a helmet-shaped 3D array of 128 transducer elements can be assembled for neurostimulation in an optimized configuration with the accommodation of an imaging aperture. Considering all acoustic losses, according to this study, for a nonhuman primate skull, the assembly of the proposed transducers was projected to produce sufficient focusing gain in two different focal positions at deep and shallow brain regions, thus providing sufficient acoustic intensity at these distinct focal points for neural stimulation. This array also has the ability to focus on multiple additional brain regions. In the work presented here, we designed and fabricated a single 15 mm diameter capacitive micromachined ultrasonic transducer (CMUT) element operating at 800 kHz central frequency with a 480 kHz 3 dB bandwidth, capable of producing a 190 kPa peak negative pressure (PNP) on the surface. The corresponding projected transcranial spatial peak pulse average intensity (I_SPPA_) was 28 Wcm^−2^, and the mechanical index (MI) value was 1.1 for an array of 128 of these elements.

## 1. Introduction

Along with numerous therapeutic applications, ultrasonic neuromodulation holds the potential to provide a non-invasive way to answer age-old questions regarding the interplay of the different regions of brain circuits responsible for numerous physical, mental, and cognitive activities. However, to efficiently study the interconnected functionalities of the brain, it is crucial to stimulate multiple regions simultaneously, to disrupt the normal functionality with a sufficient intensity of ultrasonic power, while capturing the response in real time [1,2,3].

The history of using ultrasound for treating neurological disorders is not new. However, one of the major challenges in this direction has been the transmission of ultrasound through the human skull. Therefore, craniotomy was employed in early experiments [4,5]. Later, with the development of suitable array technology, it was possible to start exploring high-intensity focused ultrasound for treating neurological disorders [6,7,8]. In addition, low-intensity ultrasound was demonstrated to be effective for neural stimulation [9]. Focused ultrasound at different intensities interacts differently with neurons, the building blocks of the human nervous system. Therefore, depending on the intensity of the applied pulse, the effect of ultrasound varies. Hence, ultrasound can be divided into three categories in terms of intensity range: high-intensity (greater than 200 Wcm^−2^), medium-intensity (between 100 and 200 Wcm^−2^), and low-intensity (less than 100 Wcm^−2^) [10]. High-intensity focused ultrasound (HIFU) is used to generate heat for tissue ablation [11,12]. Medium-intensity has the capability of non-invasively opening the blood–brain barrier and is used for delivering drugs and imaging agents. Low-intensity ultrasound is the subject of our current study, which aims at the application of neuromodulation, thanks to its ability to stimulate or disrupt neuronal activity depending on the modulation parameters [10,13].

**Figure 1:**
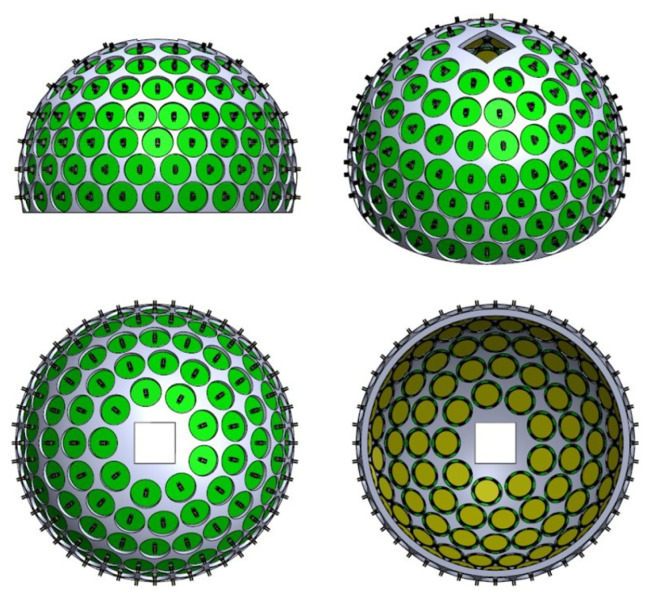
Example of envisioned 3D assembly of fabricated elemental CMUT devices in a hemispherical array with an opening for imaging (size is not scaled).

Using 3D fullwave simulations, a recent study showed for a nonhuman primate model that the position and orientation of a 3D array of 128 transducer elements with 750 kHz central frequency could be optimized to achieve a transcranial pressure gain of 5 (relative to the pressure on the transducer surface) in two separate focal points in shallow and deep brain regions simultaneously (with accommodation for an imaging aperture) [3]. The 3 dB volume of the focused beam in each position was 0.96 mm^3^. In various studies, it has been shown that an intensity (I_SPPA_) range of 25–35 Wcm^−2^ is sufficient for neurostimulation in nonhuman primates [14]. The corresponding peak negative pressure (PNP) range is 870 kPa–1 MPa. Considering a focusing gain of 5, a PNP of approximately 200 kPa on the transducer surface results in the required intensities at the focal locations. The corresponding mechanical index (MI) for this PNP at a central frequency of 750 kHz is 1.2, which is well below the FDA guideline for ultrasonic imaging (1.9) [15]. An envisioned 3D assembly of single-element devices is shown in Figure 1, following the simulation model described in detail in reference [3].

Fabrication of 3D arrays for transcranial applications has been attempted using piezoelectric transducers [16]. However, these transducers have their own intrinsic limitations, such as the use of environmentally harmful lead (Pb)-based materials, self heating, sensitivity to temperature, complex process steps for fabrication, etc. Therefore, it is crucial to explore all the possible alternatives offering a complete or partial solution to these issues. The capacitive micromachined ultrasonic transducer (CMUT) has been shown to have many advantages for a variety of applications [17,18,19,20,21]. Some of these advantages include, but are not limited to, simplified fabrication process steps, no self heating, ease of fabrication of large arrays, use of already established silicon chip manufacturing technologies, wider bandwidth, easier integration with supporting electronics, capability to realize complex device geometries, and, most importantly, batch manufacturing.

In this work, we designed, fabricated, and characterized single-element CMUTs of hexagonal shape having a 15 mm diameter circular active area that can reach approximately 190 kPa PNP at 800 kHz on the surface when modulated with a 340 kHz AC excitation with an amplitude of 40 V from the source. The corresponding MI value was 1.1. The 3 dB fractional bandwidth was 60% around a central frequency of 800 kHz. In the following section, the FEM model and obtained device parameters are explained. Later on, the fabrication process is discussed in detail in Section 3. In Section 4, the device characterization steps are discussed. The discussion in this section is divided into three subsections: atmospheric deflection conformity check, frequency response measurement in immersion, and acoustic pressure measurement in immersion.

## 2. Finite-Element-Modeling-Based Design

The inter-space between cells was set by applying the empirical design norm that the post thickness should be at least 1.5 times the plate thickness to minimize post-bending. Since the plate thickness in this design was set to 50 μm, that restricted the minimum value to be at least 75 μm. However, in addition to the bare minimum, we used 25 μm (34%) additional space as a safety margin to obtain 100 µm. This resulted in the pitch between cells being 920 μm.

The gap height is another parameter that requires careful consideration. Lowering gap height implies that a given pressure can be achieved at a lower voltage level, due to a stronger force field as a result of reduced distance. However, setting the gap height lower also means decreasing the maximum achievable pressure limit, since this restricts the space in which the plate can vibrate. The lower limit of the gap height should be set according to the expected minimum pressure output from the device. The upper limit comes from a combination of parameters. Along with the pressure consideration, the target bottom metal thickness and the gap height that can be sealed without any issue after vent etching also need to be considered. In our case, the bottom metal thickness was set to 170 nm. From previous experience, we have observed that a 600 nm gap height can be successfully sealed with 2-micron-thick silicon nitride. However, a higher gap height translates into a higher input voltage requirement to generate a given output pressure. Therefore, the gap height must be set according to the pressure requirement and fabrication limitations.

We used a commercial software package (ANSYS v19.2, ANSYS Inc., Canonsburg, PA, USA) to create a finite element model for refining the device parameters for the required performance. The model has both solid and fluid elements, to simulate the pressure field in front of the vibrating plate. For modeling the vibrating plate in the CMUT structure, we used the SOLID185 element, which is designed to have three translational degrees of freedom, unless restricted by an appropriate command (the total number of nodes for this element is 8). Then, the material parameters as shown in Table 1 were declared.

**Figure 2:**
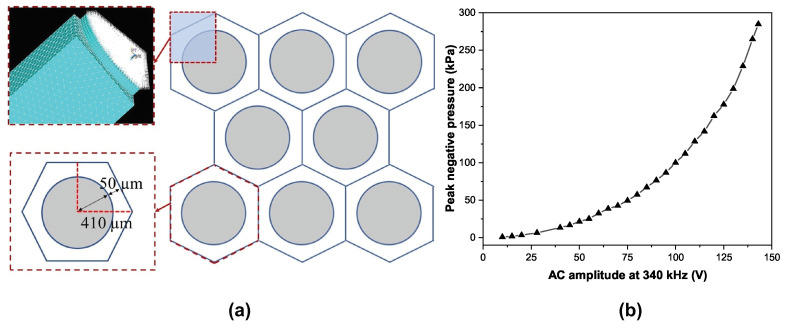
(**a**) A unit cell of simulation (**b**) peak negative pressure vs. applied AC voltage amplitude.

We used the FLUID30 element for modeling the 3D acoustic fluid part that is in contact with the solid and FLUID130 as the absorbing boundary, which extends the fluid field by absorbing the incident pressure waves. To reduce the calculation time, one-fourth of a single cell and the adjacent fluid with an absorbing boundary layer were created first (Figure 2a). Then, we performed meshing on solid and fluid parts. To simulate the electromechanical actuation, we used TRANS126 elements connected to the bottom nodes of the vibrating plate. The gap and the dielectric film thickness were entered as real constants and the electrode radius was defined by selecting the nodes of the vibrating plate in that radius. Boundary conditions were applied and the SYMM command was used to replicate the model in the X and Y directions.

To summarize, initially, we created the top inset of Figure 2a from scratch (one-fourth of the hexagon), defining it layer by layer and declaring boundary conditions, followed by meshing. Afterwards, using symmetry constraints, we created a full hexagonal cell, as shown in the bottom inset of Figure 2a. This hexagon had reflecting boundaries at the edges, which turned this structure to an infinite arrangement of hexagons. The model, at this point, was ready for calculations. As a primary step, we used static analysis to determine the collapse voltage, which was found to be 131 V, and the atmospheric deflection was 35 nm. Later, we ran a transient analysis to obtain the small signal impulse response, which allowed us to determine the central frequency and bandwidth. Following this, the displacement and pressure output for different input AC signals (frequency and amplitude) were simulated. The results are summarized in Figure 2b, where we have plotted the corresponding peak negative pressures in response to a pure AC signal of 340 kHz frequency with varying amplitude (V). The list of dimension of different parameters is shown in Table 2.

## 3. Device Fabrication

We adopted a five-mask process for the fabrication of the described devices. The flow of the fabrication steps followed a previously demonstrated anodic bonding-based process, which we reported in [22]. We used a 100 mm diameter, 700 µm thick, double-side polished borosilicate glass (Borofloat33; UniversityWafer, Inc., South Boston, MA, USA) wafer as a substrate. The main advantage of using insulator glass wafers is reduced parasitic capacitance compared to semiconductor substrates and the availability of anodic bonding for fabrication. We cleaned the glass wafer with a 3:1 solution of sulfuric acid (H_2_SO_4_-96%, J.T. Baker, Avantor Performance Materials LLC, Randor, PA, USA) and hydrogen peroxide (H_2_O_2_-30% LM, Transene Company Inc., Denvers, MA, USA), also known as the Piranha solution [23]. We kept the wafers in this solution for 15 min. After 15 min, as the evolution of gas bubbles subsided, we took out the wafer from the solution and rinsed. We finished the cleaning step by placing the wafer in a spin dryer (Model Semitool PSC-101, Shellback Semiconductor Technology, Coopersburg, PA, USA).

After drying and before spinning the photoresist, we placed the wafer in a hexamethyldisilizane (HMDS) vapor prime oven (Model YES-3TA, Yield Engineering Systems, Fremont, CA, USA) for 5 min to prime the wafer for photolithography. This step makes the surface hydrophobic immediately after drying, covered by an organic functional group, which protects the surface from gathering environmental moisture and ensures good adhesion to the photoresist.

For the first lithography step, we used a negative tone photoresist (AZ nLOF 2070, Micro-Chemicals, Ulm, Germany) spun at 3000 rpm. The aim of this step was to pattern the cavities. The thickness of the photoresist was 6.7 µm. Development with a tetramethylammonium hydroxide solution (Microposit MF-CD-26, Shipley Company, Marlborough, MA, USA) left the cavity regions exposed.

**Figure 3:**
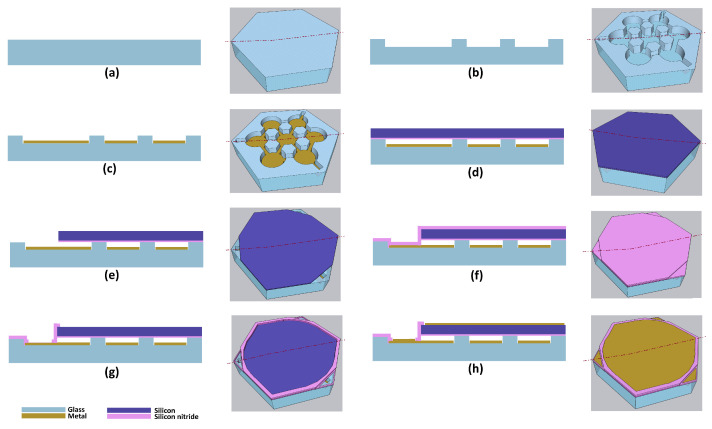
Fabrication process flow: (**a**) glass wafer cleaning, (**b**) first etching of glass wafer (RIE and BOE), (**c**) bottom metal deposition, (**d**) anodic bonding of silicon nitride deposited SOI and glass wafers, (**e**) vent etching using DRIE and RIE (after handle and BOX removal), (**f**) PECVD sealing nitride deposition, (**g**) nitride etching (RIE), (**h**) e-beam evaporation of top metal.

In the subsequent step, we used a combination of dry (reactive ion etching, RIE) and wet (buffered oxide etch, BOE) etch processes for etching the cavity regions. The reason for finishing with the wet etching was to enhance the surface smoothness. Avoiding roughness as much as possible is crucial, since any roughness present on the cavity surface eventually transfers to the metal layer (to be deposited in the subsequent step), worsening the device-to-device uniformity. The RIE (Oxford NPG80 RIE, Oxford Instruments, Abingdon, UK) recipe used a combination of sulfur hexafluoride (SF_6_—80 sccm) and oxygen (O_2_—20 sccm) at 200 W RF power at 5 °C. The etch rate of this recipe was determined to be 55 nm/min. For the wet etching, the wafer was put in a BOE bath at room temperature under a chemical hood. The etch rate of BOE was 35 nm/s. After completion of etching, the photoresist was stripped off using piranha solution and the total etch depth was verified with a profilometer (Veeco Dektak 150, Veeco Instruments Inc., Tucson, AZ, USA). The depth of the etched cavities at this point was measured as 600 nm ± 10 nm. A representative 3D drawing and corresponding cross-sectional view of this step are shown in Figure 3b.

The next step was the bottom metal deposition. We used the same photoresist (AZ nLOF 2070) for this step as before. After the development, we inserted the wafer in an oxygen plasma Asher chamber (March Instruments Inc., Concord, CA, USA) to descum (50 sccm O_2_, 250-W) for 4 min. We used an e-beam evaporator (Solution Process Development System, CHA Industries, Livermore, CA, USA) for metal deposition. At first, 20 nm chromium (Cr) was deposited at a rate of 1 Å/s, followed by the deposition of gold (Au) at a rate of 10 Å/s. During the metal deposition, the wafer holder fixture was rotated at a rate of 20 rpm. This was important for the uniformity of deposition across the wafer. The vacuum level achieved prior to the start of metal deposition was 3.5 × 10^−6^ Torr. After taking the wafer out of the chamber followed by the deposition, it was left overnight at room temperature in a N-Methyl-2-pyrrolidone (NMP; Thermo Fisher Scientific, Waltham, MA, USA) solution for liftoff. We determined the completion of liftoff through careful observation under an optical microscope. Following that, we cleaned the wafer in a heated (at 75 °C) NMP solution for 30 min. Later, after rinsing, we further cleaned the wafer with a stabilized formulation of sulfuric acid and hydrogen peroxide compounds (Nano-Strip 2X, CMC Materials EC, Inc., Fort Worth, TX, USA), followed by rinsing in a DI water bath and then drying in a spin dryer. This step marked the completion of preparation of the bottom part of the device prior to anodic bonding. This step is depicted in Figure 3c.

For the top part, we used an SOI wafer (Silicon On Insulator wafer; Biotin Crystal Company Limited, Fujian, China) with a 50 ± 1 µm device layer. The device layer was p-type doped (dopant: Boron, B), with a crystal orientation of (100) and a resistivity of 0.001–0.005 Ohm-cm. The thickness of the buried thermal oxide layer was 1.0 µm ± 5%. The handle layer had the same orientation, doping type, and dopant species as the device layer. The thickness of this layer was 250 ± 10 µm. We cleaned the wafer in piranha solution to remove any organic constituents sticking to the surface. Afterward, we performed a short BOE dipping to chemically dissolve the native oxide layer.

Following this, we inserted the wafer in a vapor deposition chamber (P5000, Applied Materials Inc., Santa Clara, CA, USA) for deposition of PECVD silicon nitride (Si_x_N_y_), which acts as a dielectric layer and also prevents electrical shorting in case of contact [24]. In the following step, the wafers were bonded in an anodic bonding chamber (SB-6E, SUSS MicroTec Inc., Corona, CA, USA) [25]. The applied force was 30 kN and the chamber pressure was maintained at 5.0 × 10^−5^ mbar. For bonding, the voltage was ramped up to 750 V in 30 min and then held there for another 30 min. Before applying the voltage, the temperature was ramped up to 350 °C in 60 min. The temperature was ramped down to room temperature after the bonding was completed. The bonded pair is illustrated in Figure 3d.

After anodic bonding, the next step was to remove the handle wafer since the service of a carrier wafer was no longer required, and we needed to vent the gases generated by the anodic bonding process and reach the bottom electrode pad locations. The handle removal was performed in two steps. The primary step was a grinding process, which left the handle wafer at a thickness of about 100 µm. After this step, the remainder was chemically etched using tetramethylammonium hydroxide (TMAH) solution (25 wt.% in water, Sigma-Aldrich, MilliporeSigma, Burlington, MA, USA). The wafer was first put in the BOE solution to remove any native oxide. Then, we transferred the wafer to a TMAH solution heated at 80 °C.

TMAH has a slow etch rate for Si and an even slower etch rate for SiO_2_. Therefore, it is practically impossible to over-etch a wafer using TMAH. The revealing of the oxide surface marked the completion of the handle wafer removal. Following this, we took the wafer out, rinsed it, and inserted it into a BOE tank for BOX removal. The etch rate of silicon dioxide (SiO_2_) in BOE (10:1) is around 55 nm/min. Etching 1 µm took approximately 18 min. We performed the etching in 1-min cycles. The completion of BOX removal was marked by changing the surface from hydrophilic to hydrophobic, which was evident by the rapid sliding of the liquid droplets past the wafer surface upon extraction from the BOE bath.

The next step was deep reactive ion etching (DRIE) of the 50 µm plate, which facilitated venting of the trapped gases that resulted from the anodic bonding step (the presence of the gases was evident from the buckling up of the plate when checked with an optical or stylus profilometer), at the same time opening the pad locations (locations for wire bonding to connect the device to the supporting PCB electronics). Another target of this etching step was to form the dicing streets separating the individual elements. We spun the same negative photoresist used in previous steps (AZ nLOF 2070), however, at a lower spin speed (2000 rpm) to increase the thickness to 8 µm. The lithography for this step required back-side alignment since the bottom electrode features and alignment marks were not visible through the 50 µm device layer.

For etching the 50 µm thick silicon plate, we used a DRIE tool (Alcatel Deep Reactive Ion Etch, Alcatel Micro Machining Systems, Annecy, France) with a SF_6_-based recipe (350-sccm SF_6_, RF power of 1800 W, and pressure of 5.5 × 10^−5^ bar). The recipe was run for 7 min, which etched the silicon approximately 45 µm (at an etch rate of 6.4 µm/min). The etch rate of the photoresist by DRIE was measured to be around 375 nm/min. The wafer was then transferred to an RIE tool (Oxford NPG80 RIE, Oxford Instruments, Abingdon, UK), which has a lower etch rate for silicon. The reason for finishing with a less aggressive etching process was to protect the underlying gold fingers, which may be damaged by DRIE in case of accidental over-etching. The etch recipe for RIE was the same as before (as we used for glass etching). For all dry etching steps, the chamber was cleaned for 10 min with oxygen plasma and conditioned by running the recipe on a test wafer for 2–4 min, before moving to the process wafer. After opening the pad locations, we removed the remaining photoresist using an oxygen plasma Asher (March Instruments Inc., recipe: 50 sccm O_2_ with an RF power of 250 W). An illustration of a vent-etched device element is shown in Figure 3e.

It is important to note that any wet processing can be detrimental at this point, since the exposed pad locations are connected to the active device locations and any liquid used at this point will leak into the cavities, affecting the device performance and reliability. After this step, the wafer was ready for sealing.

For sealing, we used a 2 µm thick low-stress PECVD nitride. After placing the wafer inside the reaction chamber (Plasma-therm 790, Plasma-therm, Saint Petersburg, FL, USA), the temperature was slowly ramped up to the deposition temperature of 350 °C. After the deposition of 2 µm, low-stress silicon nitride, the temperature was ramped down to the room temperature. This step is illustrated in Figure 3f.

Following this step, we etched the silicon nitride to expose the active areas and pad locations. Since the devices were sealed at this point (evident by the downward buckling of the plate when checked using a profilometer), it was safe to use wet processing again without running the risk of damaging the devices. The same photoresist (AZ-nLOF-2070) was used with a spin speed of 3000 rpm. We used the RIE tool (Oxford NPG80 RIE, same recipe as before) for this step. The etch rate for silicon nitride was 195 nm/min. After etching was complete, the remaining photoresist was stripped off using heated NMP (70 °C). A device element after this processing step is depicted in Figure 3g. At this point, we avoided any aggressive acid-based solvent (Nanostrip, Piranha, etc.) to avoid damage to the exposed gold fingers.

Although, it is simpler to use the same patterned photoresist for both the nitride etch and the top metal deposition, in this case, a gap was intended to be introduced between the edge of the nitride etch and later deposited metal layer. Since the lithography for the nitride etch has a step coverage of 50 μm, this may potentially lead to thinning of the photoresist along the side walls, resulting in insufficient coverage. Consequently, the nitride may be etched and the silicon may become exposed along the step locations. Afterwards, if metal is deposited with the same lithography pattern, this leaves the possibility of creating a short circuit between the top electrode and bottom electrode contact pads. To avoid this issue, an additional lithography step was introduced.

**Figure 4:**
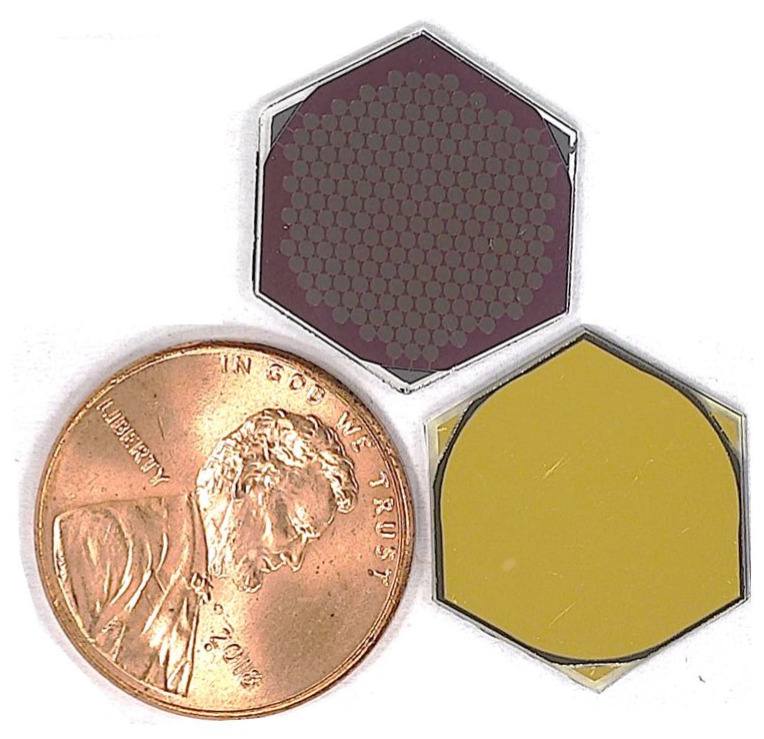
Fabricated device front (right of the penny—golden color) and back (top) side.

The final lithography for the metal deposition was performed using a thick negative photoresist (AZ-2070) with the same spin speed as the previous step. The lithography left the active area and the three corner (bottom electrode contact fingers) regions exposed for metal deposition. The metal in the active area was designed to act as the top electrode contact (also to decrease the resistivity of the top electrode), while the three triangular corner pads were designed to act as bottom electrode contacts for wire bonding. The exposure time for photolithography was 15 seconds. Then, after development, we performed a ‘descum’ for 3 min. Later, 20 nm Cr (1 Å/s) and 250 nm (10 Å/s) Au were deposited in an electron beam evaporator (the same tool that was used for bottom metal deposition). The liftoff was performed in NMP. The completion of liftoff marked the completion of the fabrication process. Figure 3h shows an illustration of a completed device element. In addition, a real image of both sides of a completed and diced device element is shown in Figure 4. A penny is placed in the image for scaling purpose.

Before dicing, we covered the wafer with a positive photoresist (Microposit S-1813, Shipley Company, Marlborough, MA, USA; at a spin speed of 2000 rpm) to protect the devices during dicing. For dicing, we used an automatic dicing saw (Model DAD 322, Disco Hi-Tec America Inc., San Jose, CA, USA). Following dicing, we cleaned the devices with acetone and isopropyl alcohol. Following this, we placed the devices on PCBs and then wire bonded (Model 74677E, West Bond, Anaheim, CA, USA) the contacts. At this point, the devices were ready for characterization.

## 4. Device Characterization

### 4.1. Atmospheric Deflection

We used a 3D optical profilometer (Wyko NT9100, Veeco Instruments Inc., Plainview, NY, USA) to check the atmospheric deflection. We cross-matched the deflection profile with the results extracted from our FEM model.

**Figure 5:**
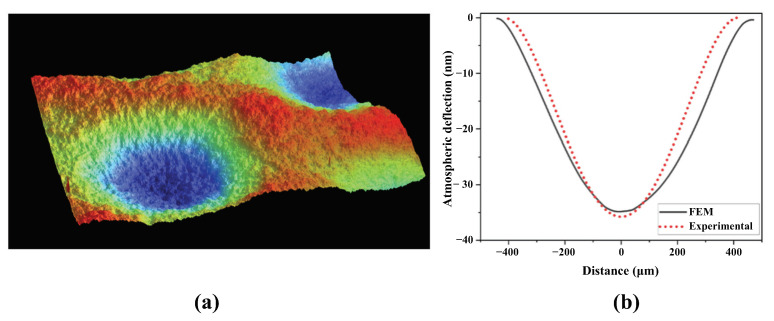
(**a**) Deflected surface of vacuum sealed cells under atmospheric pressure captured by a surface scan using an optical profilometer, (**b**) atmospheric deflection profile comparison of FEM model and experimental characterization of a single cell.

The deflection confirmed the proper sealing of the devices in vacuum. Figure 5a shows a surface scan of atmospheric deflection obtained from the Wyko surface profilometer, and Figure 5b shows the FEM calculated and experimentally measured deflection profiles. The maximum deflection in both cases was close to 35 nm (±2 nm).

### 4.2. Bandwidth Measurement in Immersion

We wire-bonded the devices to individual printed circuit boards (PCB) for acoustic characterization. We used vegetable oil as an immersion medium, as the electrically insulating property of the oil allowed us to test the devices without packaging [26]. We measured the bandwidth using a short (350 ns) pulse in oil.

**Figure 6:**
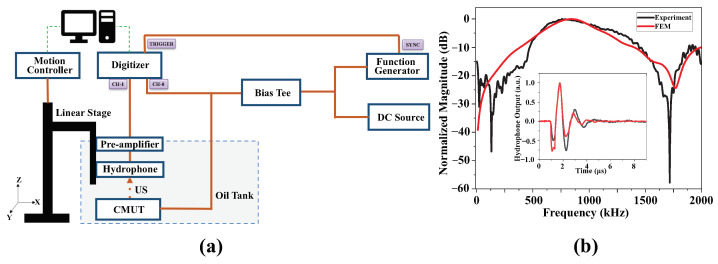
(**a**) Experimental setup for frequency response measurement in immersion, (**b**) frequency response to a 10 V 350 ns unipolar square pulse excitation in submersion, the inset plot shows the time domain signals.

The distance between the hydrophone and the device was 33 mm for this measurement. Figure 6a represents the setup used for the bandwidth measurement and Figure 6b shows the frequency response, with the inset plot showing the original output pulse recorded by the hydrophone. The lines in red represent prediction from FEM model. The 3 dB bandwidth was measured as 480 kHz, which corresponds to a 60% fractional bandwidth around 800 kHz. This is in line with our design in this therapeutic application, which requires narrowband excitation and some limited frequency tunability.

### 4.3. Acoustic Pressure Measurement by Hydrophone in Immersion

**Figure 7:**
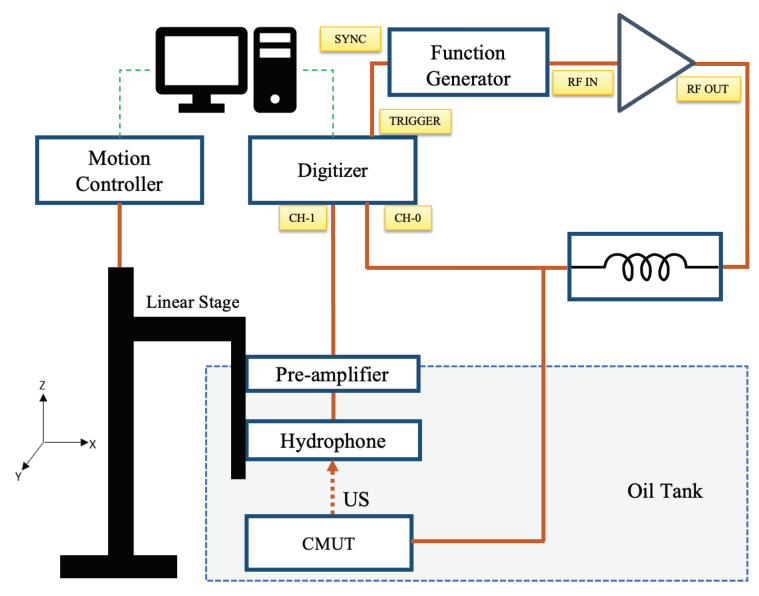
Block diagram of the experimental setup for pressure measurement in immersion.

**Figure 8:**
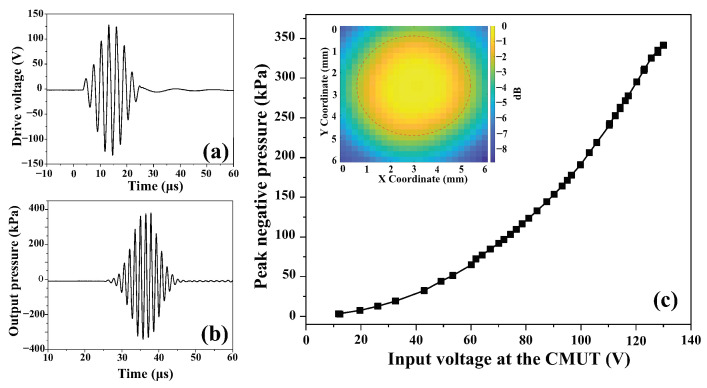
Hydrophone measurement result at a 33 mm distance from the CMUT (**a**) input signal, (**b**) obtained pressure output, (**c**) negative peak pressure profile as a function of the peak input voltage (inset shows an area scan).

**Figure 9:**
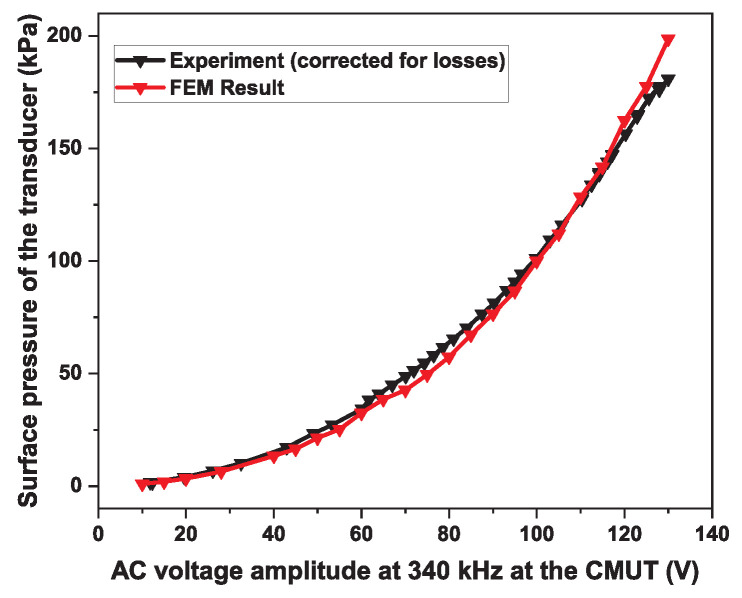
Surface peak negative pressure profile of the transducer as a function of the applied AC voltage amplitude—comparison of experimental result with FEM prediction. The voltage was measured at the CMUT input port.

To characterize the devices for output pressure, we used the experimental setup shown in Figure 7. To simplify the electronics for this application, the devices can be driven by an AC signal at half of the resonance frequency, since this is only a transmit event [27,28]. For our experiment, the AC waveform was amplified to drive the CMUTs. We synthesized the input waveform using Benchlink Waveform Builder Pro Software (version: R-X43-001-L; Keysight Technologies, Colorado Springs, CO, USA) and then generated it using a waveform generator (Model 33500B, Agilent Technologies, Santa Clara, CA, USA). We amplified the signal from the waveform generator using an RF power amplifier (Model 210L, Electronics & Innovation, Rochester, NY, USA). We used a series inductor (100 µH) as a matching circuit between the power amplifier and the CMUT. At the receiving end, we used a needle-type hydrophone (Model HNA-0400, Onda Corporation, Sunnyvale, CA, USA) along with a preamplifier (Model AG2010-20-1264 from the same manufacturer). The preamp and the hydrophone were attached to a three-axis linear stage (Model PRO165, Aerotech Inc., Pittsburgh, PA, USA). The Labview program was used to control the stage. The trigger signal was received by a high-resolution digitizer (Model PCI-5124, National Instruments, Austin, TX, USA) for scan synchronization. We also frequently used a mixed-signal oscilloscope (Model MSO-X-3024-A, Agilent Technologies, Santa Clara, CA, USA) for visualizing the signals. The frequency of the AC signal was 340 kHz. The frequency was optimized with a lower amplitude signal at the beginning of the experiment. Figure 8c shows the peak negative pressure data acquired by the hydrophone at a 33 mm distance from the device for varying input voltage amplitudes. The near field distance for a single CMUT transducer element is approximately 31 mm from the calculation (in vegetable oil medium). However, the hydrophone measured the maximum pressure level at a 33 mm distance, which is within a range of ±2 mm of the theoretical value. Therefore, the distance between the transducer and the hydrophone was set to 33 mm for the rest of the measurements. The inset of Figure 8c shows an area scan depicting the natural focus from a single element at this distance. Figure 8a shows an input signal, and Figure 8b shows the corresponding received signal converted to pressure. The received signal on the hydrophone surface at 33 mm was corrected for diffraction effect and attenuation loss to backpropagate to the CMUT surface. Figure 9 shows the peak negative pressure or rarefaction pressure from the FEM model and the corresponding experimental data for the transducer surface.

The largest value for the peak negative pressure measured was 190 kPa, which is within a range of ±10 kPa of the target pressure (200 kPa). A summary of the measured and projected parameters is given in Table 3.

## 5. Conclusions

We successfully designed and fabricated a single-element CMUT with 207 cells. We experimentally demonstrated a 190 kPa peak negative pressure on the transducer surface when excited with a 340 kHz 137 V sinusoidal signal. The device showed a bandwidth of 480 kHz around a 800 kHz center frequency (60% fractional bandwidth). The corresponding I_SPPA_ value was 28 Wcm^−2^ and MI was 1.1 (for soft tissue). The experimental pressure response data upon application of the AC signal matched closely with the FEM model. These measured characteristics of the fabricated device satisfied the specification required for the proposed multifocal neurostimulation application for nonhuman primates. The next step is to repeat the established fabrication pathway to make a 128-element array, and to package and arrange them with electrical connections in a helmet-like 3D-printed scaffold to execute multi-focusing experiments.

## Figures and Tables

**Table 1:** Material properties.

Parameters	Values
Young’s modulus of silicon	148 GPa
Poisson’s ratio of silicon	0.177
Density of silicon	2330 Kgm
Young’s modulus of silicon nitride	296 GPa
Poisson’s ratio of silicon nitride	0.27
Density of silicon nitride	3180 Kgm

**Table 2:** Parameters targeted for fabrication.

Parameters	Dimension (μm)
Cell diameter	820
Pitch between cells	920
Width of inter cell contacts	80
Thickness of top metal	0.27
Plate thickness	50
Dielectric layer thickness	0.3
Gap height	0.43
Thickness of bottom electrode metal layer	0.17

**Table 3:** Summary of measured and projected parameters.

**Measured parameters for a single fabricated element**			
Center frequency	Active area diameter	Surface peak negative pressure, PNP	Peak AC voltage amplitude at 340 kHz
(kHz)	(mm)	(kPa)	(V)
800	15	190	130
**Projected numbers ^1^ for the 3-D array of 128 elements according to reference [3] **			
Gain	PNP	Intensity, I_SPPA_	MI
	(kPa)	(Wcm^−2^)	
5	950	28	1.1

^1^ The values are for transcranial condition, at two simultaneous focal positions, and assuming soft tissue as the focusing medium.

## Data Availability

Data are available within the article.

## Abbreviations

The following abbreviations are used in this manuscript:

BOE	Buffer Oxide Etch
CMUT	Capacitive Micromachined Ultrasonic Transducers
DRIE	Deep Reactive Ion Etching
FEM	Finite Element Method
HIFU	High-Intensity Focused Ultrasound
MI	Mechanical Index
PECVD	Plasma Enhanced Chemical Vapor Deposition
PNP	Peak Negative Pressure
RIE	Reactive Ion Etching
TMAH	Tetramethylammonium Hydroxide

## References

[1] Yoo S.-S., Bystritsky A., Lee J.-H., Zhang Y., Fischer K., Min B.-K., McDannold N.J., Pascual-Leone A., Jolesz F.A. (2011). Focused ultrasound modulates region-specific brain activity. NeuroImage.

[2] Tufail Y., Yoshihiro A., Pati S., Li M.M., Tyler W.J. (2011). Ultrasonic Neuromodulation by Brain Stimulation with Transcranial Ultrasound. Nat. Protoc..

[3] Jones R.M., Caskey C.F., Dayton P.A., Oralkan O., Pinton G.F. (2022). Transcranial Neuromodulation Array with Imaging Aperture for Simultaneous Multifocus Stimulation in Nonhuman Primates. IEEE Trans. Ultrason. Ferroelectr. Freq. Control.

[4] Tsivgoulis G., Alexandrov A.V. (2016). Ultrasound in Neurology. CONTINUUM Lifelong Learn. Neurol..

[5] Fry F.J., Ades H.W., Fry W.J. (1958). Production of Reversible Changes in the Central Nervous System by Ultrasound. Science.

[6] Thomas J.-L., Fink M.A. (1996). Ultrasonic Beam Focusing through Tissue Inhomogeneities with a Time Reversal Mirror: Application to Transskull Therapy. IEEE Trans. Ultrason. Ferroelectr. Freq. Control.

[7] Sun J., Hynynen K. (1999). The Potential of Transskull Ultrasound Therapy and Surgery Using the Maximum Available Skull Surface Area. J. Acoust. Soc. Am..

[8] Jung H.H., Kim S.J., Roh D., Chang J.G., Chang W.S., Kweon E.J., Kim C.-H., Chang J.W. (2015). Bilateral Thermal Capsulotomy with MR-Guided Focused Ultrasound for Patients with Treatment-Refractory Obsessive-Compulsive Disorder: A Proof-of-Concept Study. Mol. Psychiatry.

[9] Tyler W.J., Tufail Y., Finsterwald M., Tauchmann M.L., Olson E.J., Majestic C. (2008). Remote Excitation of Neuronal Circuits Using Low-Intensity, Low-Frequency Ultrasound. PLoS ONE.

[10] Kubanek J. (2018). Neuromodulation with Transcranial Focused Ultrasound. Neurosurg. Focus.

[11] Kennedy J.E., Ter Haar G.R., Cranston D. (2003). High Intensity Focused Ultrasound: Surgery of the Future?. BJR.

[12] Ter Haar G. (2001). Acoustic Surgery. Phys. Today.

[13] Plaksin M., Kimmel E., Shoham S. (2016). Cell-Type-Selective Effects of Intramembrane Cavitation as a Unifying Theoretical Framework for Ultrasonic Neuromodulation. eNeuro.

[14] Verhagen L., Gallea C., Folloni D., Constans C., Jensen D.E., Ahnine H., Roumazeilles L., Santin M., Ahmed B., Lehericy S. (2019). Offline Impact of Transcranial Focused Ultrasound on Cortical Activation in Primates. eLife.

[15] Radjenovic S., Dörl G., Gaal M., Beisteiner R. (2022). Safety of Clinical Ultrasound Neuromodulation. Brain Sci..

[16] Kim Y., Maxwell A.D., Hall T.L., Xu Z., Lin K.-W., Cain C.A. (2014). Rapid Prototyping Fabrication of Focused Ultrasound Transducers. IEEE Trans. Ultrason. Ferroelectr. Freq. Control.

[17] Haller M.I., Khuri-Yakub B.T. (1996). A Surface Micromachined Electrostatic Ultrasonic Air Transducer. IEEE Trans. Ultrason. Ferroelectr. Freq. Control.

[18] Brenner K., Ergun A., Firouzi K., Rasmussen M., Stedman Q., Khuri–Yakub B. (2019). Advances in Capacitive Micromachined Ultrasonic Transducers. Micromachines.

[19] Krenkel M., Stolz M., Koch S.G., Kupnik M. (2019). CMUT with Mechanically Coupled Plate Actuators for Low Frequencies. J. Micromech. Microeng..

[20] Gerardo C.D., Cretu E., Rohling R. (2018). Fabrication and Testing of Polymer-Based Capacitive Micromachined Ultrasound Transducers for Medical Imaging. Microsyst. Nanoeng..

[21] He C., Zhang B., Xue C., Zhang W., Zhang S. (2021). Wafer-Bonding Fabricated CMUT Device with Parylene Coating. Micromachines.

[22] Yamaner F.Y., Zhang X., Oralkan Ö. (2015). A Three-Mask Process for Fabricating Vacuum-Sealed Capacitive Micromachined Ultrasonic Transducers Using Anodic Bonding. IEEE Trans. Ultrason. Ferroelectr. Freq. Control.

[23] Schmidt H.G. (2022). Safe Piranhas: A Review of Methods and Protocols. ACS Chem. Health Saf..

[24] Liu L., Liu W., Cao N., Cai C. (2013). Study on The Performance of PECVD Silicon Nitride Thin Films. Def. Technol..

[25] Knowles K.M., Van Helvoort A.T.J. (2006). Anodic Bonding. Int. Mater. Rev..

[26] Javanaud C., Rahalkar R.R. (1988). Velocity of Sound in Vegetable Oils. Fett/Lipid.

[27] Yamaner F.Y., Olcum S., Oguz H.K., Bozkurt A., Koymen H., Atalar A. (2012). High-Power CMUTs: Design and Experimental Verification. IEEE Trans. Ultrason. Ferroelectr. Freq. Control.

[28] Yamaner F.Y., Olcum S., Bozkurt A., Koymen H., Atalar A. Design and Implementation of Capacitive Micromachined Ultrasonic Transducers for High Power. Proceedings of the 2011 IEEE International Ultrasonics Symposium.

